# Supplementary figures and images for: Large-Scale Heat-Tolerance Screening and Genetic Diversity of Pea (Pisum sativum L.) Germplasms
Source: Plants (Basel). 2022 Sep 21;11(19):2473. doi: 10.3390/plants11192473 (PMC9573610; doi:10.3390/plants11192473)

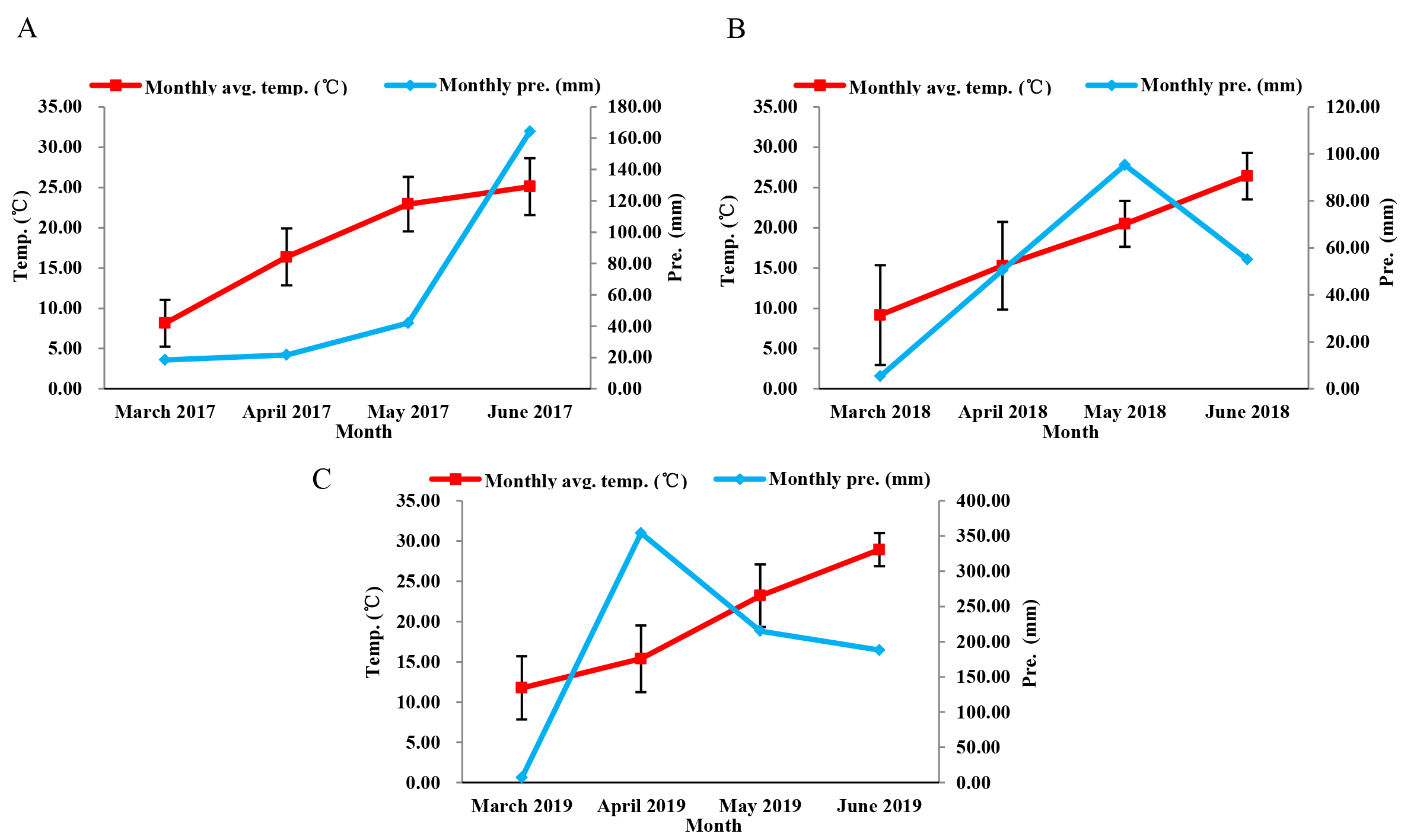

Supplement: Supplementary file 1 [file plants-11-02473-s001.zip › plants-1887571-supplementary/Supplementary Material/Figure S1.tif]

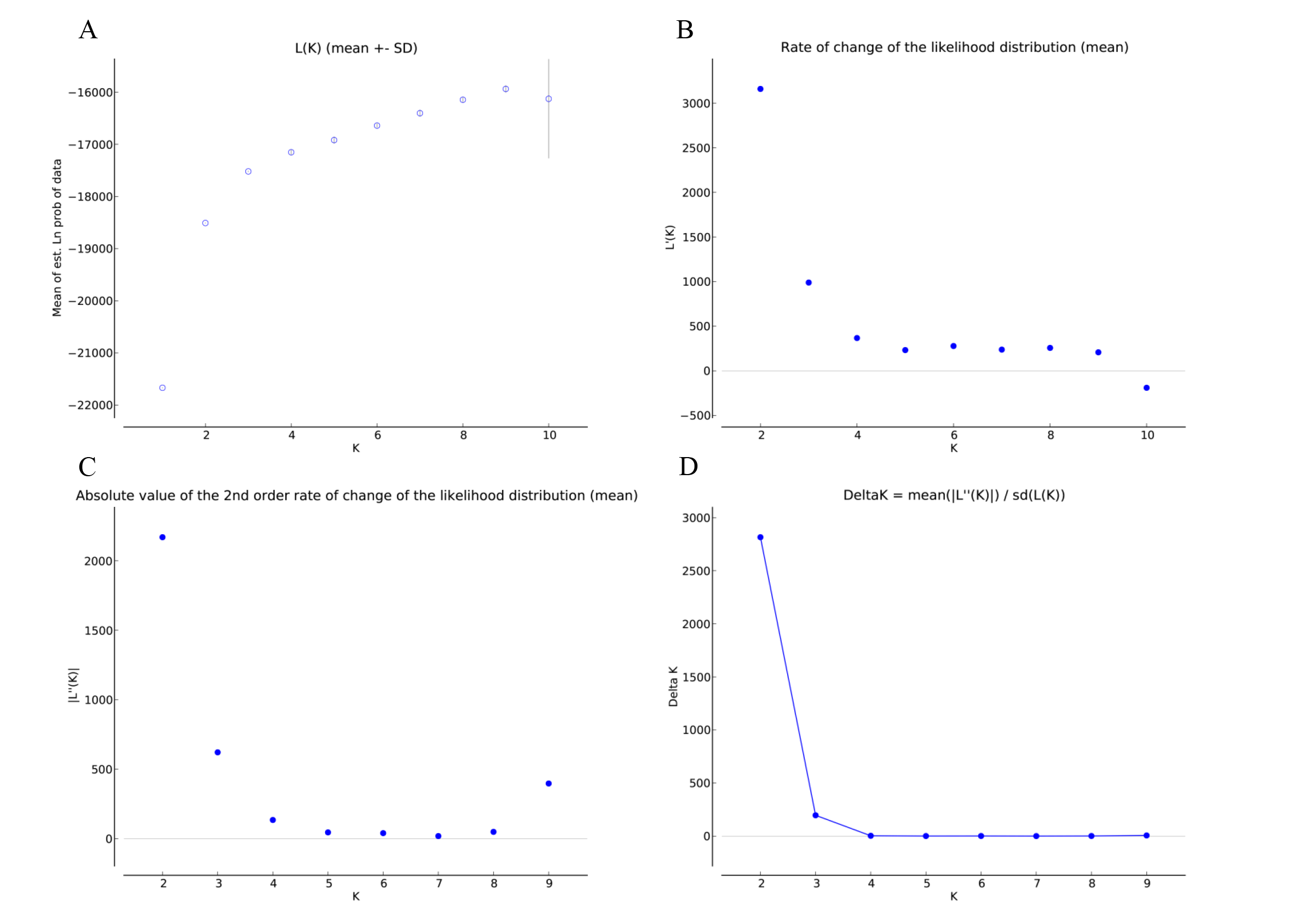

Supplement: Supplementary file 1 [file plants-11-02473-s001.zip › plants-1887571-supplementary/Supplementary Material/Figure S2.tif]

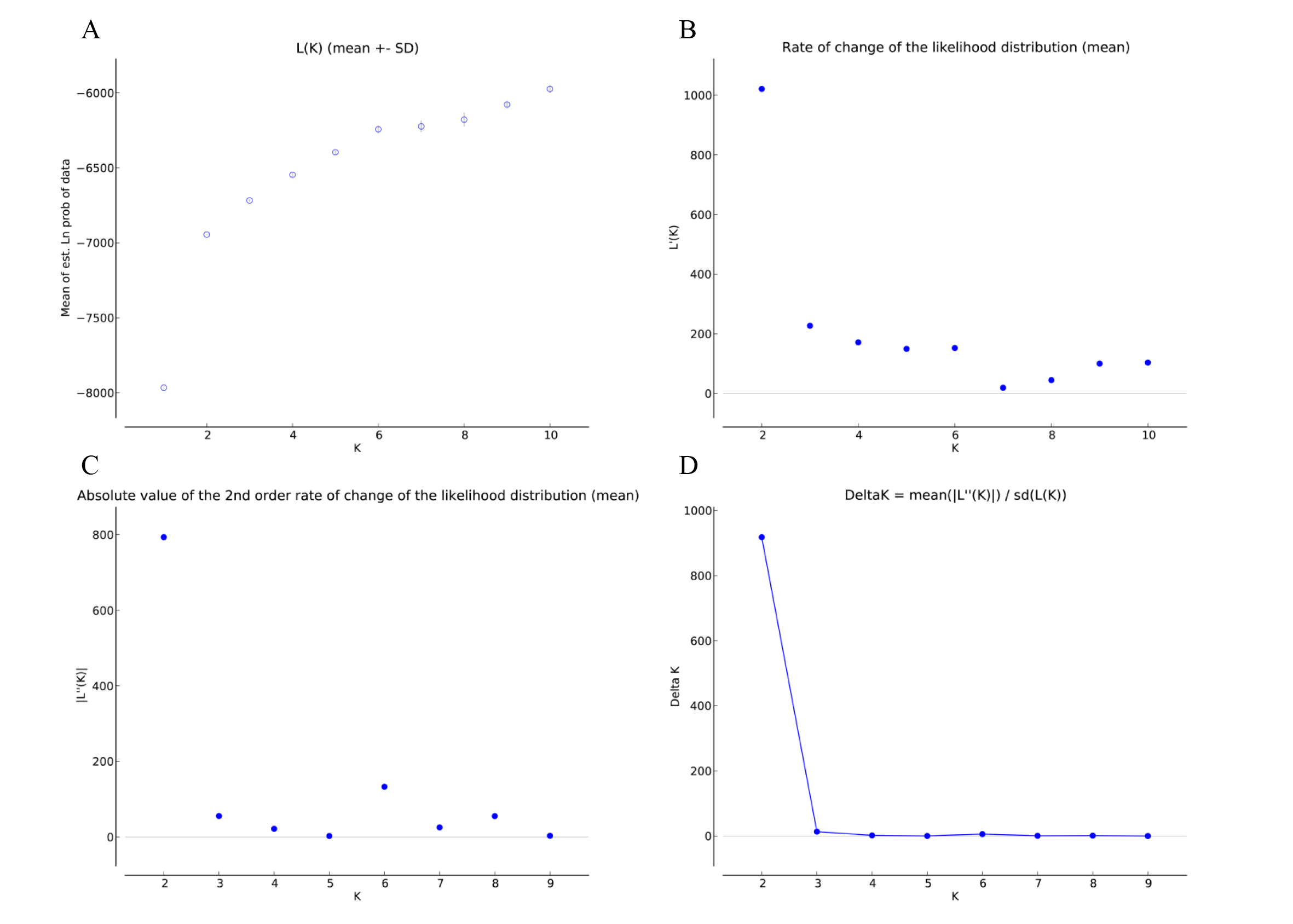

Supplement: Supplementary file 1 [file plants-11-02473-s001.zip › plants-1887571-supplementary/Supplementary Material/Figure S3.tif]
